# Scalable, Robust and Highly Productive Novel Convecdiff Membrane Platform for mAb Capture

**DOI:** 10.3390/membranes12070677

**Published:** 2022-06-30

**Authors:** Mario Grünberg, Kim B. Kuchemüller, Katrin Töppner, Ricarda A. Busse

**Affiliations:** Sartorius Stedim Biotech GmbH, August-Spindler-Strasse 11, 37339 Goettingen, Germany; mario.gruenberg@sartorius.com (M.G.); kim.kuchemueller@sartorius.com (K.B.K.); katrin.toeppner@sartorius.com (K.T.)

**Keywords:** bioprocessing, downstream processing, antibody purification, protein A chromatography, membrane chromatography, process intensification, single-use chromatography

## Abstract

The recombinant monoclonal antibody capture step represents the current bottleneck in downstream processing. Protein A resins are diffusion-limited chromatography materials which require low flow rates to achieve a binding capacity above 30 g L^−1^ with the result of low productivity. Here, we present a novel chromatography membrane combining superior binding capacities with high flow rates for high productivity while achieving comparable product quality as state-of-the-art protein A resins. Further, we demonstrate full scalability of this convecdiff technology with experimental data demonstrating suitability for bioprocessing at different scales. This technology results in more than 10-fold higher productivity compared to Protein A resins, which is maintained during scale up. We demonstrate the influence of residence times, feed titers and the cleaning regime on productivity and indicate optimal utilization of the convecdiff membrane based on feed titer availability. The underlying high productivity and short cycle times of this material enable the purification of monoclonal antibodies with 10-times less chromatography material used per batch and utilization of the membrane within one batch. Provided in disposable consumables, this novel technology will remove column handling in bioprocesses and resin re-use over multiple batches.

## 1. Introduction

Processes to purify recombinant monoclonal antibodies (mAb) for therapeutic treatment are well established in the market. However, continuous improvements to reduce patient risk and the cost for such treatments are implemented. Over the past decade, efforts to improve efficiency have focused on the upstream side of increasing titers [1]. These efforts result in a shift of the bottleneck towards the downstream side [2]. The downstream process typically comprises three chromatographic purification steps. Specifically, the mAb capture step which still depends on protein A resin in a packed-bed column format has obvious short comings, such as high diffusional resistance leading to long process time, re-use over multiple batches to make it economical which, in turn, requires extensive cleaning and validation efforts, as well as tedious column packing [3]. Considerable efforts are being made to identify alternative chromatographic materials which bypass these limitations while providing comparable product quality and support therapeutics which are affordable to more people [4].

To date, available chromatographic matrices can be clustered into two categories based on their dominant mass transport capabilities. The first category comprises porous chromatographic resins. Here, the dominant mass transport mechanism is based on diffusion into the porous structure. Inside of resins, the effective pore diffusion is slow and the distances to be covered are comparatively large (~30–50 µm). This leads to process operations at low flow rates and high residence times which result in low productivity (10–30 g L^−1^ h^−1^) of this unit operation [5]. Materials of this design can be denoted as diffusive materials.

The second category contains purely convective materials such as membranes, fiber-beds or monoliths. The predominant transport mechanism in these materials is based on convection as the binding sites are presented at the surface of the convective pores. This structure supports good accessibility of the ligand but comes with a trade-off between binding capacity and pressure drop because both values are tuned by the pore size while showing contrariwise behavior. Pore sizes that ensure acceptable binding capacities for a protein A-functionalized purely convective material (>30 g L^−1^) are in the sub-micron range (e.g., 0.3 µm). This forces the user to either accept high pressure drop and increased fouling propensity, or low binding capacity [6].

We present a new generation of chromatographic materials that combines structural and performance aspects of resins and purely convective materials. This new convecdiff membrane contains a high binding gel phase with short diffusional path length (2–3 µm) and large convective pores for fast transport to the gel phase [7]. This combination offers robust and scalable high binding capacities at short residence times. In addition, the large convective pore sizes ensure low fouling propensity and easy cleanability, as well as high permeability, allowing for bed heights of about 4 mm or more with low pressure drops.

In this article, we demonstrate that the novel convecdiff membrane with a protein A ligand provides typical binding capacities > 30 g L^−1^ for a set of tested Fc-containing molecules at residence times of 12 s, as well as robust performance and product quality over 200 cycles. In addition, we show scalability of three different membrane volumes by comparing critical process parameters (CPP) and critical quality attributes (CQA). We further prove comparability of the CQA of an mAb purified with the convecdiff membrane and a state-of-the-art resin which showed the same high product quality for both materials. Beyond this, we analyzed the impact of different parameters, such as feed titer, flow rate and sanitization frequency on productivity and recommend an optimal combination of these parameters to maximize productivity (g L^−1^ h^−1^).

In summary, the novel convecdiff membrane is a viable, robust and scalable alternative to protein A resins used for mAb capture. It provides a magnitude increase in productivity compared to diffusion-limited chromatography resin. Specifically, molecules with a low feed titer benefit from the high flow rates. However, as productivity is also influenced by cycle time the obtained productivity is lower compared to high feed titers. When high feed concentrations are obtained the effect of DBC_10%_ significantly increases and the flow rate during the load phase should be accordingly adjusted to achieve maximum productivity. In this study, optimal flow rates at maximum productivity were determined for various feed concentrations. Further improvement in productivity can be achieved by reducing the regeneration frequency to every 7th cycle. This will further increase productivity by 11.8 ± 6.5%, beyond this no further significant improvement was observed.

## 2. Materials and Methods

### 2.1. Buffers, Reagents and Monoclonal Antibodies

Chemicals used for buffer preparation were purchased from Carl Roth (Karlsruhe, Germany) and buffer constitutions are listed in Table 1. Buffers and recipes used in this study are subject to internal platform approach.

All recombinant human monoclonal antibodies were expressed in Chinese hamster ovary (CHO) cells using standard cell culture techniques (stirred bioreactor). The cultivations were performed in 5 L Biostat^®^ bioreactors (Sartorius Stedim Biotech, Goettingen, Germany) in batch mode for 14 days. Cell clarification was performed in a two-step depth filtration using Sartorius Sartoclear DL20 and DL60 (Sartorius Stedim Biotech, Goettingen, Germany) with subsequent sterile filtration using Sartopore 2 XLG (Sartorius Stedim Biotech, Goettingen, Germany). Table 2 summarizes the antibodies used in this study.

### 2.2. Protein A Chromatography Devices

Protein A chromatographic devices used were novel Sartorius convecdiff membrane prototypes with a membrane volume (MV) of 1.2 mL (capture of 4 different mAbs), 10 mL and 70 mL (proof of scalability) with bed heights of about 4 mm, as well as a HiTrap MabSelect SuRe column from Cytiva (Uppsala, Sweden) with a column volume (CV) of 1 mL (comparison adsorber versus resin).

### 2.3. Protein Concentration and Monomer Determination by Size Exclusion HPLC

Protein concentrations and monomer/aggregate levels of harvested cell culture fluid (HCCF) and purified samples were determined by analytical high-performance size exclusion chromatography (SEC-HPLC) using a TSKgel G3000SWXL-column (30 mm ID × 7.8 cm) from Tosoh (Griesheim, Germany) with an UltiMate 3000 HPLC System from ThermoFisher Scientific (Dreieich, Germany). The HPLC system was operated at 1 mL·min^−1^ with PBS as mobile phase applying 10 µL of sample. The elution profile was monitored at λ = 280 nm using the systems spectrophotometer. Elution peak area was converted to protein concentration using a standard curve generated with purified material. Aggregate levels were determined as a ratio of peak areas of the early-eluting aggregate peak(s), late-eluting fragment peak(s) and the monomer peak.

### 2.4. Dynamic Binding Capacity Measurements

DBC is defined as the maximum amount of target protein that can be loaded onto a stationary phase without causing unnecessary loss, measured under realistic experimental conditions. Dynamic binding capacity (g of mAb per L of membrane/resin) was determined for chromatographic devices (see Section 2.2) using an ÄKTA Avant 150 chromatography system from Cytiva (Uppsala, Sweden) in two different ways.

(1) For the cycling studies, the DBC was determined using HCCF. The device was equilibrated and then loaded with the HCCF containing the mAbs until overloading (at 12 s residence time). The flowthrough was fractionated in 1-mL portions to identify the volume when the stationary phase was fully saturated and mAb breakthrough occurred. A DBC (total bound mAb) was calculated for the amount of HCCF loaded where monomer was below the level of detection in the breakthrough. The protein concentration of the feed and the breakthrough-fractions were determined with SEC-HPLC using a standard curve generated with purified material.

(1) $$\mathrm{DBC}=\frac{V_{0\%}\times C_{0}}{V_{membrane}}$$

where *V*_0%_ is the volume where no mAb can be measured in the flowthrough fraction (L), *C*_0_ is the mAb concentration (g·L^−1^) in HCCF and *V_membrane_* is the volume of the membrane bed in the chromatographic devices. Determination of DBC with HCCF reflects the real process due to physicochemical mAb interactions with impurities, as well as the competition and hindrance of accessibility to the ligand.

(2) For the other experiments, DBC_10%_, defined as the amount of protein loaded at 10% breakthrough (g of mAb per L of membrane/resin) was determined. Purified protein load was adjusted to pH 7.0 ± 0.2. The device was equilibrated and then loaded with protein (feed concentration: C_Feed_ ∼1.0 g L^−1^) until the stationary phase was saturated. The exact protein concentration of the feed was determined by offline A280 measurement using the Unchained Labs Little Lunatic (Pleasanton, CA, USA).

(2) $${\mathrm{DBC}}_{10\%}=\frac{\left(V_{10\%}-V_{0}\right)\times C_{0}}{V_{column/membrane}}$$

where *V*_10%_ is the volume at which 10% breakthrough was observed, *V*_0_ = system void volume (L), *C*_0_ is the mAb concentration (g L^−1^) and *V_column_*_/*membrane*_ is the volume of the resin of the membrane in the chromatographic devices. The breakthrough was determined at a residence time of 12 s for convecdiff membrane prototypes and 4 min for HiTrap MabSelect SuRe.

### 2.5. Determination of Productivity

The productivity of the utilized chromatography devices for the purification of the different mAbs was calculated according to:

(3) $$\mathrm{PR}=\frac{m_{mAb}}{V_{column/membrane}\times \;t_{c}}$$

where PR (g L_MV_^−1^ h^−1^) is the productivity, *m_mAb_* (g) is the average eluted mass of monoclonal antibody, *V_column_*_/*membrane*_ (L) is the volume of the resin/membrane in the chromatographic devices and *t_c_* (h) is the average cycle time over the whole process.

### 2.6. Protein A Capture Chromatography from Harvested Cell Culture Fluid

Capture of monoclonal antibodies from HCCF was conducted with convecdiff membrane with a membrane volume of 1.2 mL for cycling studies with different mAbs and Cytiva HiTrap MabSelect SuRe (see Section 2.2) as a comparison with regard to different critical process parameters (CPP) for one mAb using an ÄKTA Avant 150 chromatography system or Sartorius MU RCC system (Pompey, France). Chromatography was performed with buffers and chromatography recipes mentioned in Table 1 and Table 3.

The load was calculated as 80% of the DBC_10%_ measured at a residence time of 12 s for the membrane or as 80% of the DBC_10%_ measured at a 4 min residence time for the resin depending on the HCCF titer. The load density was conservatively chosen to achieve the desired number of cycles without product loss. Elution pools were collected from 100–100 mAU (using ÄKTA spectrophotometer with a 2 mm path length at λ = 280 nm). Step yield was determined using the mass of the product in the load and pool (both determined by SEC-HPLC).

### 2.7. Host Cell Protein, hcDNA and Leached Protein A Measurements

Host cell protein (HCP) concentrations were measured using the CHO HCP ELISA Kit3G F550-1 kit from Cygnus Technologies (Southport, NC, USA). Host cell DNA concentrations were measured using the Quant-iT PicoGreen dsDNA assay kit from Thermo Fisher (Dreieich, Germany). The log-reduction-value (LRV) of both impurities was determined by means of the decadic logarithm of the quotient of impurity concentration in the feed and the impurity concentration in the elution fraction. Leached protein A was measured using the Protein A ELISA Kit (9000-1) from Repligen (Waltham, MA, USA). The values listed refer to ng protein A per mg mAb. All assay kits utilized according to the manufacturer’s instructions and analyzed in an Infinite M Nano+ plate reader from Tecan (Maennedorf, Switzerland). Every 20th elution fraction was collected and analyzed regarding different CQA (critical quality attributes) and CPP.

## 3. Results

### 3.1. Robustness of New Convecdiff Membrane—Evalution of Chromatographic Performance and Product Quality Attributes over 200 Cycles

The convecdiff membrane was evaluated for its capability to run 200 robust and reproducible cycles. The cycling study included detection of UV traces, pressure drops, elution peak symmetry and critical quality attributes of the product such as yield, monomer content, HCP and hcDNA removal, as well as protein A ligand leaching. Further, to demonstrate the robustness of the convecdiff membrane the same chromatography protocol (see Table 3) was used for all four mAbs.

**Figure 1:**
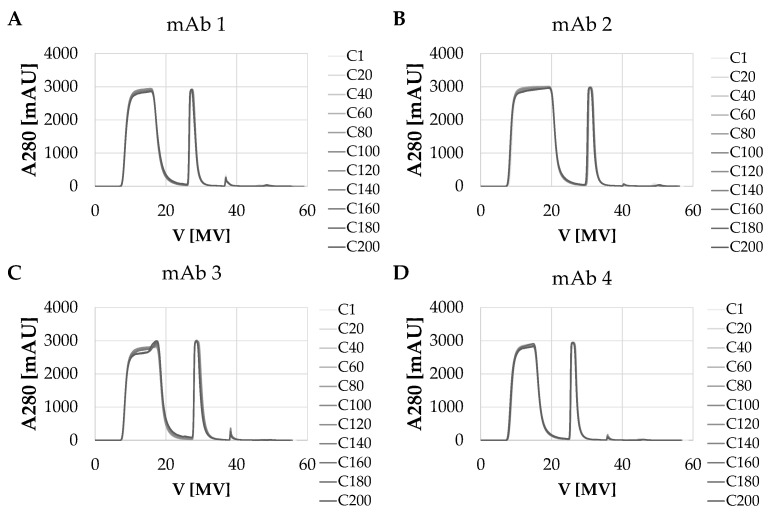
Overlays of UV traces of 200 bind and elute cycles of mAbs 1–4 (**A**–**D**) using novel convecdiff membrane. Shown is an overlay of every 20th cycle for each experiment.

Figure 1 shows the overlays of UV adsorption at 280 nm for all four mAbs over the 200 cycles. During the load phase of the feed, unbound components flow through the convecdiff membrane resulting in high UV absorbance after approximately 8 MV. In the following wash step, unbound components are flushed out of the membrane leading to a decrease in the UV signal to zero (at ~27 MV). For elution of the bound mAb, the pH is decreased resulting in release of the target molecule from the membrane which, again, results in an increase in the UV signal at approximately 30 MV. After the elution block, the regeneration solution is applied on the membrane resulting in release of non-eluted mAbs and sticky impurities. A small peak during regeneration appeared in each cycle. Subsequently, the membrane was re-equilibrated to flush out the regeneration buffer and to restore optimal conditions for the next bind and elute cycle of mAb.

For each of the four mAbs, the intended number of mAb capture cycles was achieved with very high reproducibility. The peak shapes of the UV signals are extremely consistent. No significant peak broadening or shifts were observed over the whole process time. For only mAb 3, a slight change in the UV signal was observed during the loading. This was caused by degradation of the HCCF during the process time and resulted in a decrease in yield. The overall process time for 200 cycles was around 34 h.

**Figure 2:**
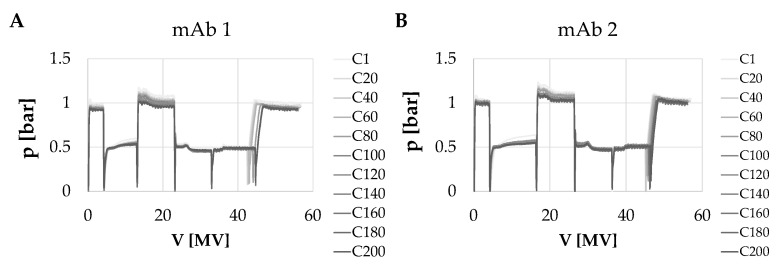

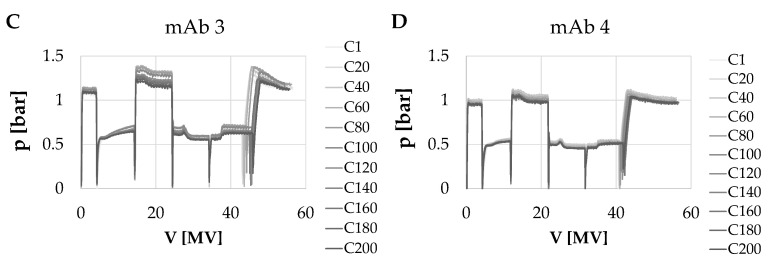
Overlays of pressure traces of 200 bind and elute cycles of mAbs 1–4 (**A**–**D**). Shown is an overlay of every 20th cycle for each experiment.

In addition, the pressure behavior over 200 cycles was detected. The highest observed pressure drops were detected during the wash step for which a flowrate of 10 MV min^−1^ was applied and when the stationary phase was saturated. Here, maximum pressure between 1.1–1.4 bar (0.11–0.14 mPa; see Figure 2) was detected. Overall, the pressure behavior of the convecdiff membrane showed only small variations within 0.2 bar indicating that the membrane was not affected by considerable fouling under the applied conditions over 200 cycles.

**Figure 3:**
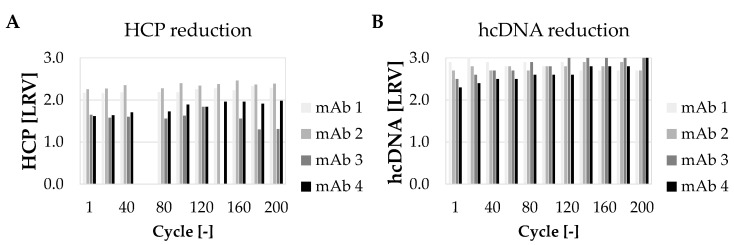
Capability of convecdiff membrane to reduce (**A**) HCP and (**B**) hcDNA (log reduction value; LRV) over 200 cycles. Shown is LRV for every 20th cycle (cycle 60 for HCP was not measured). HCCF values for each mAb: mAb 1 (HCP = 54004 ppm, hcDNA = 2320 ppm); mAb 2 (HCP = 48,158 ppm, hcDNA = 4873 ppm); mAb 3 (HCP = 20,233 ppm, hcDNA = 2360 ppm) and mAb 4 (HCP = 10,305 ppm, hcDNA = 4750 ppm).

Additionally, the CQA of the product were analyzed, including HCP removal, hcDNA removal, protein A ligand leaching and high and low molecular weight (HMW and LMW) species. As shown in Figure 3, consistent results were achieved for the 200 cycles for HCP and hcDNA removal for each HCCF. mAb 3 and 4 show lower log reduction values (LRV) due to lower HCP and hcDNA content in the HCCF. As described earlier, mAb 3 HCCF was not stable over the processing time which also results in a decrease in the LRV at cycles above 130. However, for hcDNA depletion, no effect was observed. These data confirm a consistent removal of both HCP and hcDNA over 200 cycles for four different mAbs demonstrating the cycling capability and stability of the membrane adsorber considering that no optimization of the purification protocol was performed.

Further process- and product-related parameters were summarized in Table 4. For three of the four mAbs, all analyzed CPP and CQA values showed extremely high consistency over the 200 cycles. mAb 3 showed consistent values until cycle 130. After this, the HCCF started to degrade and form aggregates. Therefore, two values are given in Table 4 for this mAb to demonstrate that, until cycle 130, the same high CPP and CQA values were achieved with the convecdiff membrane.

Purification with the convecdiff membrane resulted in extremely high yields for three of the four mAbs. MAb 4 showed lower yields, which can be explained by higher elution pH (pH 3.2) compared to the other mAbs (pH 2.9). An improvement in the yield for this mAb could be achieved by further protocol optimization. Further, the membrane showed very low capability to introduce aggregation and fragment formation resulting in a remarkably high monomer concentration in the elution fractions. Furthermore, the elution volumes and mAb concentration in the elution showed no significant change over the 200 cycles (5–6 MV; 5–6.5 g L^−1^; depending on HCCF). Another important parameter is ligand leaching of the protein A ligand from the membrane during purification. The analysis of this value in the product showed very low levels, consistently below 3.4 ppm. Compared to usual protein A resins, the leached ligand is at a comparable level but in contrast and despite cleaning with caustic after every single cycle, there was no noticeable decrease in binding capacity as typically seen with resins [9].

The underlying calculated productivities were exceptionally high which is a result of very short cycle times (average cycle times of 10–11 min) and very low stationary phase of the chromatography material (1.2 mL MV). Variations between the four experiments are due to different feed concentrations and binding capacities, which directly influence the cycle time.

### 3.2. Scalability of Convecdiff Membrane Implemented in Different Device Sizes

To demonstrate the scalability of the novel convecdiff membrane, the membrane was implemented in standard membrane capsules with a bed height of 4 mm for all scales. Scalability was investigated using three different membrane adsorber sizes with membrane volumes of 1.2 mL, 10 mL and 70 mL. The purification protocol from Table 3 was applied, and HCCF containing mAb 1 was loaded to the membrane adsorbers. The available HCCF originated from different cultivations and, therefore, contained slightly different mAb concentrations. The impurity and aggregate levels were in a similar range.

**Figure 4:**
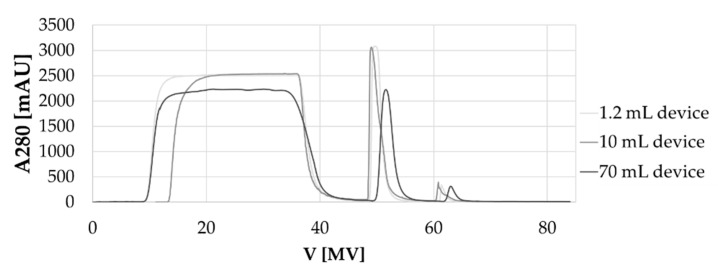
Overlays of UV traces from exemplarily bind and elute cycles of mAb 1 implemented into three different device sizes.

As shown in Figure 4, the chromatograms showed very similar peak shapes. Slight variations resulted from the different HCCF. The mAb concentration of the feed for the 10 mL device was slightly higher compared to the other two tested sizes and, therefore, the load phase was shorter resulting in a shorter UV curve. The 70 mL adsorber required a pilot scale chromatography skid. In comparison to the benchtop system used for the 1.2 mL and 10 mL device, the larger skid had different void volumes, UV sensors and other signal smoothing factors. As a result, the UV signal for the 70 mL device during load and wash phase, as well as the elution peak appears slightly shifted to the right and with a lower intensity. However, this is a common challenge during scale up.

The analyzed CPP and CQA values are summarized in Table 5. The overview of process-related parameters and product quality attributes show very high comparability. Elution concentrations of the mAbs range from 4.1 to 5.0 g L^−1^, elution volumes from 4.4 to 5.1 MV and yields from 98.0 to 99.4%. These data support high scalability of the membrane when implemented into ready-to-use devices at a fixed bed height of 4 mm.

### 3.3. Comparability of Product Quality Attributes and Critical Process Parameters after Purification Using the Convecdiff Membrane and a Standard Resin

To compare CPP and CQA between different stationary phases, mAb 1 was purified with a standard protocol over the convecdiff membrane and a resin. For the resin, the recommended protocol from the manufacturer was chosen (Table 3). The volume of the stationary phase used was 1.2 mL for the membrane and 1 mL for the column.

**Figure 5:**
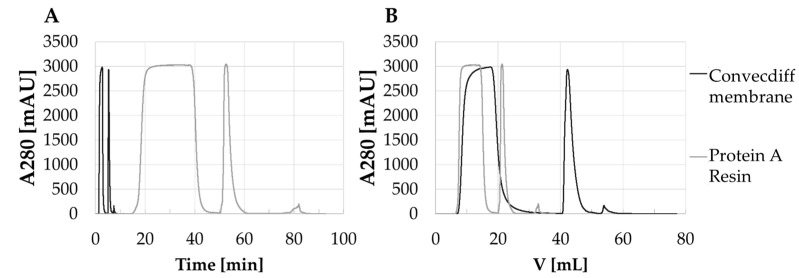
Comparison of (**A**) cycle time and (**B**) volumes of one single cycle of mAb capture with protein A resin versus convecdiff membrane adsorber.

The membrane showed an average DBC_10%_ of 42.9 g L^−1^ at 12 s residence time compared to the resin were a DBC_10%_ of 30.4 g L^−1^ was achieved at 4 min residence time. Based on this first evaluation of the DBC_10%_ for each chromatographic matrix, the resin/membrane was loaded with 80% of DBC_10%_ to simulate common practice in the industry. This resulted in a comparable yield for both materials in the range of 94.5–96.8% in the elution fraction. In addition, impurity removal and leached ligand for both materials were within a comparable range (see Table 6). Slightly higher LRV for hcDNA removal was detected for the membrane. However, a significant difference of both materials can be observed in the average productivity. Here, the membrane shows 14-fold higher productivity compared to the resin. As described above, this high productivity is mainly achieved by the fact that the membrane reaches similar binding capacities with a 20-times shorter residence time (higher flow rate) resulting in very short cycle times (see Figure 5A). Figure 5B shows the buffer consumption of both materials used to perform one purification cycle, demonstrating that the membrane required 1.74 L per g of purified mAb while the resin required 1.21 L g_mAb_^−1^. The higher buffer consumption of the membrane compared to the resin is a result of the properties of the stationary phase and the device (higher volume of stationary phase 1.2 mL membrane vs. 1 mL resin, not considered).

### 3.4. Factors Influencing Productivity of the Convecdiff Membrane

After performance testing of the convecdiff membrane and experimental demonstration of the high productivity of the membrane, factors influencing productivity were analyzed to determine further optimization potential of the productivity in order to identify the sweet-spot for process implementation.

#### 3.4.1. Influence of Residence Time (Flow Rate), Binding Capacity and Feed Titer (Cycle Time) on Productivity

**Figure 6:**
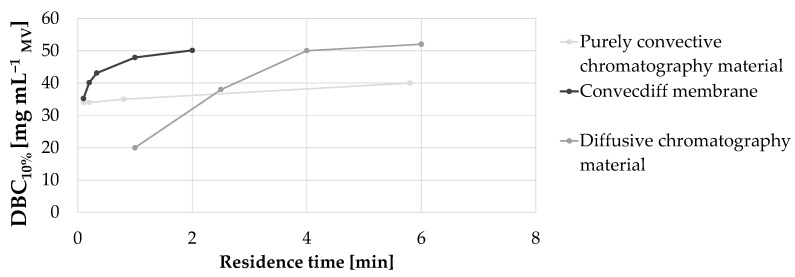
DBC_10%_ as a function of residence time for several commercially-available materials and the convecdiff membrane prototype. The convecdiff membrane prototype (1.2 mL MV and 4 mm bed height), as well as some of the other membranes/materials were tested internally. All other data points are taken from competitor [6,10,11].

First, the dynamic binding capacity of the convecdiff membrane was experimentally determined at varying residence times (by varying the flow rate) from 12 s to 2 min (see Figure 6). This was compared with DBC_10%_ data for a purely convective material and a diffusive chromatography material [10,11]. As shown in Figure 6, similar to diffusion-limited resins, the convecdiff membrane showed an increase in DBC_10%_ when residence time was increased. A dynamic binding capacity of 50.1 mg mL^−1^ at 2 min residence time and 35.2 mg mL^−1^ at 0.1 min residence time was reached using this prototype. At about 1–2 min residence time, the plateau of maximum DBC_10%_ was achieved while resins reach the plateau at around 4–6 min of residence time. The purely convective material showed a very limited dependence of residence time on DBC_10%_.

As expected, the purely convective chromatography material, like a fiber material without diffusive regions, is not limited by diffusion and, therefore, showed a very limited dependence of DBC_10%_ on residence time. The convecdiff and diffusive materials have diffusive regions and, therefore, exhibit a residence-time dependent binding capacity. This is caused by mass transport based on diffusion, which is a slow process, and is dominant especially inside the material, which increases by lowering the residence time or increasing the diffusive distance (particle size) [6].

**Figure 7:**
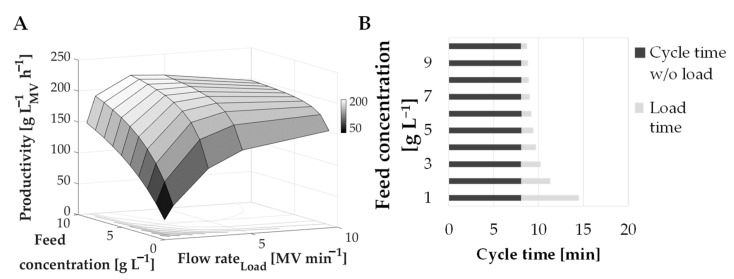
(**A**) Productivity as a function of product titer and flow rate during load for the convecdiff membrane prototype. Data points are based on internally investigations and calculations. (**B**) Cycle time in dependency to feed concentration.

Based on this relationship between residence time and DBC_10%_, the productivity was calculated as a function of the flow rate as well as product titer (see Figure 7A). The product mass and therefore the feed volume which was required to reach the DBC_10%_ was calculated according to the recipe in Table 3. Additionally, a regeneration step after each cycle was assumed.

Although highest DBC_10%_ are obtained at high residence times and low flow rates, this combination does not automatically lead to the highest productivity, as shown in Figure 7A. The highest productivity with 219.4 g L^−1^_MV_ h^−1^ for a feed concentration of 10 g L^−1^ was achieved at 3 MV min^−1^. In general, with decreasing titer the optimum was achieved with increasing flow rate (productivity of 201.5 g L^−1^_MV_ h^−1^ with 5 MV min^−1^ flow rate and 5 g L^−1^ feed concentration, as well as 152.1 g L^−1^_MV_ h^−1^ at 10 MV min^−1^ flow rate and 1 g L^−1^ feed concentration).

**Figure 8:**
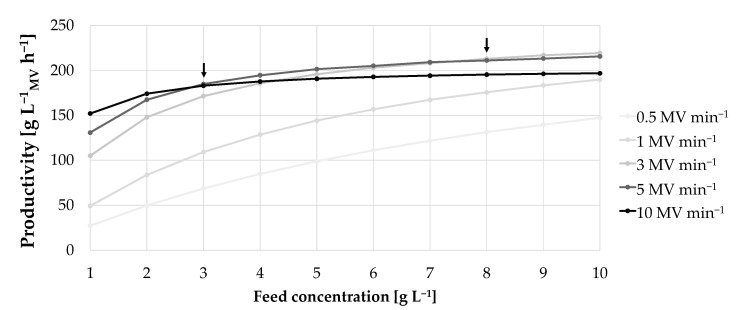
Productivity as a function of feed concentration at different flow rates during the load phase.

Thus, for low feed concentrations, the maximum productivity was achieved with high flow rates (see Figure 7A). This results from a decreasing cycle time when feed concentrations increase which influence the productivity (see Figure 7B). Apparently, cycle time had a stronger effect on productivity than DBC_10%_, as DBC_10%_ did not compensate the associated residence time. Below a feed concentration of 2 g L^−1^, the respective cycle time is so high that a higher DBC_10%_ would increase cycle time even more and, thus, the positive effect on productivity is neglectable. From 3 g L^−1^ upwards the DBC_10%_ starts to have a stronger effect, as shown in Figure 8.

In summary, for a feed concentration of ≥3 g L^−1^, the flow rate should be decreased from 10 MV min^−1^ to 5 MV min^−1^ to maximize productivity. A further reduction in the flow rate to 3 MV min^−1^ is recommended for feed concentration of ≥8 g L^−1^. Due to a reduction in flow rate, the DBC_10%_ is increased, which has a greater effect as the load phase for high feed concentrations is very short (see Figure 7B). This indicates that if high feed concentrations are available the focus should be on optimizing the load time versus DBC_10%_.

#### 3.4.2. Influence of Regeneration Frequency and Duration on Productivity

The regeneration phase in the protocol considerably contributed to the cycle time and required additional volumes of re-equilibration buffer to prime the membrane for the next cycle. Therefore, the influence of regeneration frequency and duration on productivity was investigated.

**Figure 9:**
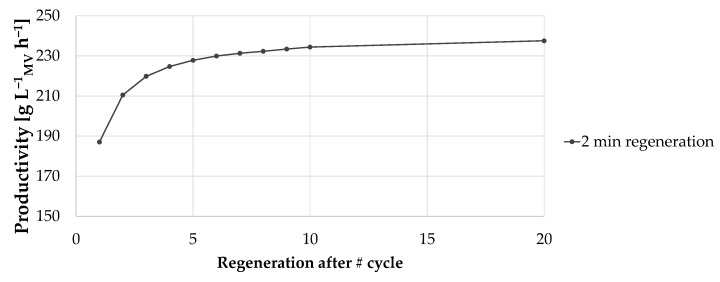
Productivity in dependency to the regeneration frequency, exemplary shown for low feed titer. Black dots constitute a regeneration duration of 2 min.

As shown in Figure 9, reducing regeneration frequency showed an effect on productivity only if regeneration was reduced within a limited number of cycles. A reduction beyond every 7th cycle is not recommended as the effect on productivity is negligible. The duration time of the regeneration phase had no significant effect on productivity (not shown). Due to the large pore structure of the convecdiff membrane, the regeneration interval can be reduced and, thus, productivity increased.

The findings for further improvement in productivity were summarized in Table 7. A reduction in regeneration frequency from each cycle to every 7th cycle for the optimal conditions detected in Section 3.4.1, leads to an additional increase in productivity of 11.8 ± 6.5%. A reduction in regeneration frequency can be achieved, e.g., when the number of impurities in the HCCF is comparatively low [12,13,14].

Impurity levels and composition of HCCFs vary depending on the design of upstream processing (e.g., cell line, fermentation conditions) and cell clarification [12,13,14]. In the case of an expected or measured low impurity level, a reduction in the regeneration frequency should be considered. Applying a HCCF comprising a low level of impurities, e.g., from a perfusion cultivation [15], leads to reduced risk of fouling, enables a reduction in the regeneration intervals and, thus, leads to an increase in productivity.

#### 3.4.3. Productivity during Scale Up

Another important factor for process implementation of a technology is that it is easily scalable. Scalability on an experimental level was demonstrated in Section 3.2. However, scalability of productivity is also important during scale up as optimization of productivity at small scale is only valuable if kept constant over different process scales.

**Figure 10:**
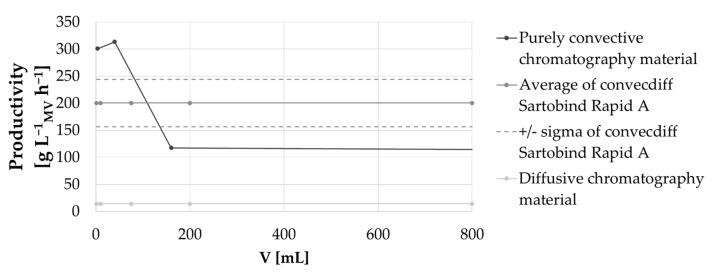
Productivity as a function of membrane/column volume for different categories of chromatographic materials. For the convecdiff membrane, an average value from this study is shown, as well as the range of the obtained productivities.

As shown in Figure 10, productivity is also slightly influenced during scale-up. Only diffusion-limited chromatography materials and the novel convecdiff membrane showed constant productivity during scale up. The purely convective fiber material showed a decrease in productivity over different device scales [16]. This lack of scalability might result from a small pore size which requires a further adaptation of the bed height and an increase in the frontal bed surface to accommodate increasing pressure drops when MV and device size are increased.

## 4. Discussion

In this study, the robustness of the novel convecdiff membrane for the capture of mAbs from HCCF was demonstrated. For four different mAbs, the CCP and CQA were analyzed, demonstrating that there is no membrane-related decrease in performance over 200 cycles. As the convecdiff membrane consists of a novel structure (mass transport that is not primarily driven by diffusion but also not purely by convection), as well as very low bed heights in adsorbers, evaluation of the robustness of the membrane in a housing cannot be tested in the same way that packed beds in columns with HETP or plate number would be [17]. The preservation of binding capacity (measured with yield), the pressure behavior, impurity removal and leached ligand, as well as elution volume were, therefore, assessed for membrane lifetime over 200 cycles. These parameters are indicators of robustness and fouling propensity of the protein A stationary phases [18,19].

The parameters analyzed for the four different mAbs varied within a very narrow range over the entire number of cycles. The integrity of the stationary phase was maintained over 200 cycles. This revealed that the fouling propensity of the membrane adsorber is very low. The fouling process was minimized by adding a short regeneration/cleaning step after each bind and elute cycle. This also indicated that the ligand was stable to repeated mild caustic exposure. Protein A ligands tend to undergo hydrolysis during caustic exposure which also typically leads to loss of binding capacity and yield [9,20]. The lowest yields in this study using the convecdiff membrane were observed for the experiments with the highest load density. Besides this, yield was constant over 200 cycles. Slight decreases in yield in the study of mAb 3 were related to changes in the feed constitution. Between cycle 130 and 140, the feed showed an increase in aggregate formation, indicating that the holding time of the HCCF was exceeded under the applied conditions. A lower yield rate for mAb 4 compared to the other mAbs seemed to be a result of the chosen elution conditions (elution mAbs 1–3: pH 2.9 ± 0.1; mAb 4: pH 3.2 ± 0.1). Since pH reduction is the main driver for the release of a mAb from protein A, the lower pH elution buffer resulted in better elution performance and, thus, higher yields. Some mAbs aggregate at lower pH (this did not occur in this study, see Table 4), therefore, a higher pH was tested.

Scalability of the membrane implemented into different ready-to-use device sizes was demonstrated considering both CQA and CPP in respect to 10 mL and 70 mL membrane volume (and 200 mL MV, data not shown). This allows for process development at a small scale, as well as its scale-up or process validation (scale-down). The higher buffer consumption, as well as elution volume and concentration resulted from a non-optimized protocol. In addition, an effect on buffer consumption and elution volume comes from the mass transport in the membrane which does not solely rely on diffusion, the device design of the membrane housing, different fluid dynamic properties of the stationary phase and a not-preferable low ratio of stationary phase to void volumes (chromatography system, tubing connections). Assuming a back mixing of 100 mL for a 2 L column or a 70 mL membrane, the back mixing ratio for the adsorber is disadvantageous for the membrane. However, this effect is leveled out for larger membrane volumes.

Compared to resin beads as the stationary phase in chromatography, the convecdiff membrane showed very similar CPP and CQA results. The investigated parameters differ slightly in terms of impurity reduction and ligand leaching. It should be emphasized that, for the chosen HCCF (mAb 1), the binding capacity of the membrane adsorber was significantly higher at a lower residence time compared to the resin. The convecdiff mass transfer in the protein A membrane allowed very short residence times without substantial losses in binding capacity. As a result, a 14-fold higher productivity of the convecdiff membrane compared to the standard resin was achieved. This is an increasingly important aspect in the purification of biopharmaceuticals, enabling substantial cost, time and space savings, as well as process intensification of future processes [21,22,23].

However, productivity is influenced by different factors such as cycle time, feed concentration and dynamic binding capacity. Providing that the novel convecdiff membrane also showed a dependency of DBC on residence time, productivity can be maximized by determining the best combination of those three influencing factors. The modulation of productivity for the convecdiff membrane revealed that low feed titers exhibit the highest productivities at high flow rates as, in this study, cycle time had the strongest influence on productivity. For feed titers larger than 3 g L^−1^_,_ flow rates in the range of 5 MV min^−1^ revealed the highest productivities. High feed titers (beyond 8 g L^−1^) cause a very short load phase, therefore, a reduction in flow rate by concurrently improving DBC_10%_ is preferable to maximize productivity. We also demonstrated that a reduction in the regeneration frequency will support further productivity increases. However, a reduction in the regeneration frequency is highly dependent on the feed conditions and contaminant levels, and this will have an impact on fouling of the membrane and might reduce the lifetime of the membrane. A first indication of membrane fouling is an increase in pressure. For HCCFs with low contaminant levels, the convecdiff membrane still shows constant performance over 300 consecutive cycles (data not shown). The large convective pores in the membrane and the low propensity of the material for unspecific binding allow for a reduction in the regeneration regime.

It is recommended to optimize productivity for each feed to best utilize the convecdiff membrane when implemented into processes. The higher the productivity is, the better will be the utilization of membrane capacity and time. In addition, we demonstrated constant productivity over different device sizes at a fixed bed height, which enables easy scalability.

In summary, this novel convecdiff membrane technology will solve the bottleneck of the mAb capture step in downstream processes of monoclonal antibodies. Provided in ready-to-use devices, it will reduce hands-on time to prepare packed bed columns. The short cycle times enable lifetime utilization of the membrane within one batch.

## 5. Conclusions

State of the art protein A materials still represent the workhorse as they offer the best compromise between high regulatory acceptance and specificity despite high costs. Certainly, in terms of costs and process intensification, they leave considerable room for improvement. Some efforts are being made to improve productivity and reduce the cost of resins using a multicolumn approach. However, this makes the mAb capture step very complex and prone to risks. In both cases, relatively large volumes of resins to purify a batch are required and the columns are re-used over multiple batches to lower cost which, in turn, adds a high risk of a bioburden event to the process and the product.

With this new technology, two main pain-points of the industry can be eliminated: first, packed bed chromatography and potential failure of it are avoided; second, re-use of those columns to make them economically viable. In addition, this new ready-to-use and disposable alternative will provide cost benefits in low number processes, where resins are typically underutilized, such as clinical scale processes and molecules with a low yearly demand. Human resources can focus on high-value tasks since column packing and cleaning validation are obsolete. Further, from a regulatory perspective, this technology will allow savings in processes as bed failure events, bioburden issues and cross-contamination between batches will be minimized.

## Figures and Tables

**Table 1:** Buffers utilized for the chromatographic experiments.

Buffer	Phase	Ingredients	pH
PBS	(Re-)equilibration, wash	1 × PBS [8]	7.4 ± 0.2
Elution-buffer	Elution	0.1 M acetic acid, 150 mM NaCl	2.9 ± 0.1 ^1^ 3.2 ± 0.1 ^2^
Reg-buffer	Regeneration/cleaning	0.2 M NaOH	>12.5

^1^ elution pH mAbs 1–3. ^2^ elution pH mAbs 4.

**Table 2:** Monoclonal antibodies and their properties.

Molecule	Class	pI	MW (kDa)
mAb1	Antibody IgG1	8.36	145.41
mAb2	Antibody IgE	7.33	146.50
mAb3	Antibody IgG1	8.09	146.53
mAb4	Antibody IgG1	8.68	145.23

**Table 3:** Chromatography recipes for one cycle of mAb capture with convecdiff membrane versus resin.

	Convecdiff Membrane		Protein A Resin	
Phase	V (MV)	Flowrate (MV min^−1^)	V (CV)	Flowrate (CV min^−1^)
Equilibration	5	10	5	0.5
Load (g L^−1^)	34.4	5	24.3	0.3
Wash	12	10	6	0.5
Elution	12 ^1^	5	12 ^1^	0.5
Regeneration	9–10 ^2^	5	2	0.2
Re-Equilibration	15–16 ^3^	10	6	0.5
av. cycle time (min)	-	9.6	-	100.4

^1^ fractionation of elution peak from 100–100 mAU at λ = 280 nm. ^2^ hold until pH ≥ 12.3, then additional 4 MV. ^3^ hold until pH ≤ 7.5, then cycle ends.

**Table 4:** Overview of process-related parameters and product quality attributes.

	mAb 1	mAb 2	mAb 3	mAb 4
Titer (g L^−1^)	3.12	2.25	3.50	4.30
DBC (g L^−1^)	42.2	41.8	54.3	49.9
Load ^1^ (g L^−1^)	32.8	32.6	42.0	38.7
Yield (%)	95.7 ± 1.3	96.3 ± 2.6	92.1 ± 1.6 ^2^ 88.3 ± 7.7 ^3^	87.3 ± 1.7
Monomer (%)	>99.5	>99.5	99.0 ^2^ 97.5 ^3^	>99.5
HCP reduction (LRV)	2.2 ± 0.1	2.4 ± 0.1	1.5 ± 0.2 ^2^	1.8 ± 0.1
hcDNA reduction (LRV)	2.8 ± 0.1	2.8 ± 0.1	2.8 ± 0.2 ^2^	2.6 ± 0.2
Protein A leached (ppm)	2.7 ± 0.7	-^4^	- ^4^	2.8 ± 0.3
av. Productivity (g L^−1^ h^−1^)	167.7	160.3	204.7 ^1^ 196.2 ^2^	206.9

^1^ Load 77.5% of DBC. ^2^ for cycles 1–130. ^3^ for cycles 1–200. ^4^ was not measured.

**Table 5:** Overview of process-related parameters and product quality attributes.

	1.2 mL Device	10 mL Device	70 mL Device
Load * (g L^−1^)	25.0	25.0	25.0
Yield (%)	98.0 ± 2.5	98.8 ± 0.3	99.4 ± 1.9
HCP reduction (LRV)	2.2 ± 0.2	-	2.3 ± 0.1
hcDNA reduction (LRV)	2.8 ± 0.2	-	2.8 ± 0.1
Elution volume (MV)	4.4	4.1	5.1
av. Productivity (g L^−1^ h^−1^)	141.4	142.5	143.4

* Load density was set to 25 g L^−1^ to achieve the desired cycle number.

**Table 6:** CPP and CQA of mAb 1 purified with convecdiff membrane and standard resin.

	Convecdiff Membrane	Protein A Resin
DBC_10%_ (g L^−1^)	42.9 ± 0.8	30.4 ± 0.5
Residence time (min)	0.2	4.0
Yield (%)	94.7 ± 0.2	96.4 ± 0.4
HCP reduction (LRV)	2.2 ± 0.2	2.3 ± 0.1
hcDNA reduction (LRV)	2.9 ± 0.2	2.3 ± 0.1
Protein A leached (ppm)]	2.7 ± 0.7	6.7 ± 0.3
av. productivity (g L^−1^ h^−1^)	203.6	14.1

**Table 7:** Highest productivity to be achieved in dependency of titer, flow rate during load, DBC_10%_ and CIP frequency.

Regeneration	Productivity (g L_MV_^−1^ h^−1^)		
	Titer = 1 g L^−1^ (Load = 10 MV min^−1^, DBC_10%_ = 35.2 mg mL^−1^)	Titer = 5 g L^−1^ (Load = 5 MV min^−1^, DBC_10%_ = 40.1 mg mL^−1^)	Titer = 10 g L^−1^ (Load = 3 MV min^−1^, DBC_10%_ = 43.1 mg mL^−1^)
Each cycle	152.1	201.5	219.4
Every 7th cycle	181.5	248	271
